# Case Report: Spontaneous coronary artery dissection and ventricular fibrillation cardiac arrest –treatment dilemma of whether to place an implantable cardioverter defibrillator

**DOI:** 10.3389/fcvm.2026.1754406

**Published:** 2026-02-12

**Authors:** Holden Caplan, Sarah Leventhal, Brian Barr

**Affiliations:** University of Maryland Medical Center, Baltimore, MD, United States

**Keywords:** CCTA, spontaneous coronary artery dissection, coronary computed tomography angiography, fibromuscular dysplasia, ICD, SCAD

## Abstract

**Background:**

Spontaneous coronary artery dissection (SCAD) is a rare, non-atherosclerotic cause of acute coronary syndrome (ACS) predominantly affecting young women, often associated with fibromuscular dysplasia (FMD). Though increasingly recognized, it remains underrepresented in literature.

**Case summary:**

We present a case of a young woman with ACS and ventricular arrhythmia leading to cardiac arrest due to SCAD and discuss clinical decision-making regarding secondary prevention ICD placement. This patient was discharged without an ICD. Unfortunately, she was rehospitalized in the setting of persistent vertigo and subsequently diagnosed with fibromuscular dysplasia (FMD) in the setting of a carotid artery dissection. She remained stable and was discharged with close cardiology and neurology follow-up for management of FMD.

**Discussion:**

There is no definitive evidence for or against placement of a secondary prevention ICD in patients who present with ventricular arrhythmias in the setting of SCAD due to FMD. This case highlights the complexity of SCAD management, particularly regarding ICD placement for secondary prevention. Further research into long-term outcomes, especially in SCAD cases associated with genetic vasculopathies like FMD, is needed.

**Conclusion:**

This case highlights the challenges of ICD decision-making in SCAD with systemic vasculopathy and emphasizes the importance of guideline-directed conservative management.

## Introduction

Spontaneous coronary artery dissection (SCAD) is a rare, non-atherosclerotic, non-iatrogenic cause of acute coronary syndrome (ACS) that involves the spontaneous formation of a hematoma in the tunica media of the coronary artery, causing luminal compression and ischemia (1). Cases of SCAD are often associated with a new diagnosis of fibromuscular dysplasia (FMD), a lifelong multifactorial illness that may lead to recurrent arterial dissections (2). We present a case of a young woman with ACS and ventricular arrhythmia leading to cardiac arrest due to SCAD and discuss the clinical decision making regarding secondary prevention ICD placement.

## Case summary

A 45-year-old African American woman with a past medical history notable only for episodic dizzy spells presented to a community hospital emergency room with acute onset chest pain while at work as a schoolteacher. The pain radiated to her back and was associated with left arm numbness. There were no identifiable precipitating risk factors present on review of systems and no family history of sudden cardiac death.

On arrival, she experienced progressive non-bloody, non-bilious emesis. Her vital signs were stable, and initial lab work revealed a creatinine of 1.0 mg/dL, potassium 3.7 mEq/L, magnesium 1.9 mg/dL, HbA1C 5.6%, LDL 84 mg/dL, and a negative pregnancy test.

An initial 12-lead ECG showed normal sinus rhythm without signs of acute ischemia.

Shortly after, she became diaphoretic and experienced seizure-like activity with no palpable pulse. Cardiopulmonary resuscitation was initiated, and she was defibrillated twice for ventricular fibrillation. Return of spontaneous circulation was achieved after 20 min. High-sensitivity troponin-I level was elevated to 19 ng/L. The patient regained consciousness but continued to report chest pain, concerning for ongoing ischemia. A post-resuscitation ECG revealed an anterior ST-elevation myocardial infarction (STEMI).

**Figure 1:**
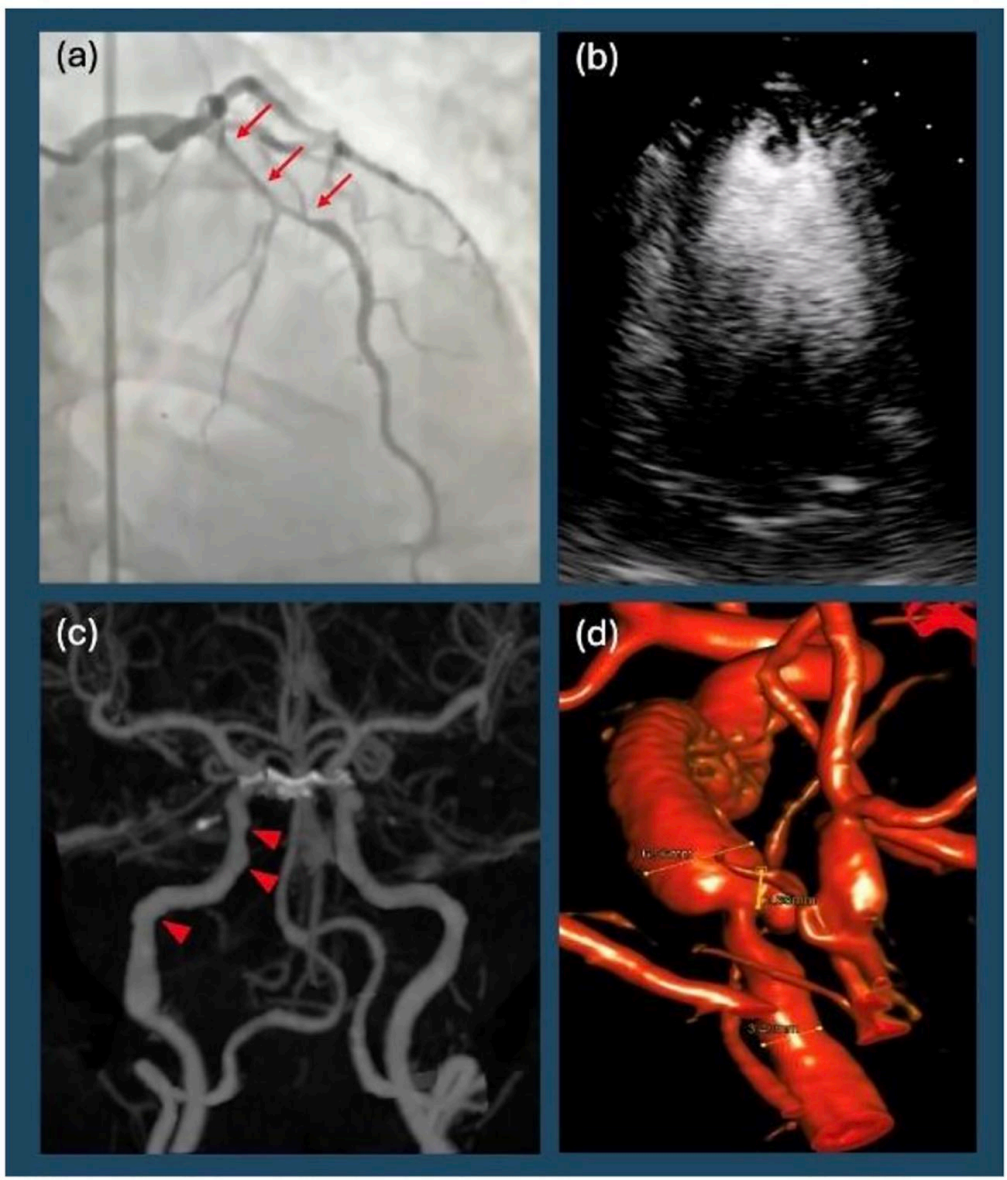
Diagnostic imaging. **(a)** Patient's initial left heart catheterization showing diffuse, long, smooth stenosis of the mid left anterior descending artery (arrows), consistent with Type II spontaneous coronary artery dissection. **(b)** Transthoracic echocardiogram with contrast showing apical left ventricular thrombus in addition to reduced ejection fraction. **(c)** Computer Tomographic angiography of the head and neck revealing focal beaded irregularity of right internal carotid artery (arrowheads) consistent with fibromuscular dysplasia. **(d)** Digital subtraction angiography showing right distal internal carotid artery dissection with partially thrombosed pseudoaneurysm.

Transthoracic echocardiography (TTE) revealed severely reduced systolic dysfunction with a LV ejection fraction (LVEF) of 15%–20% and left anterior descending (LAD) territory akinesis. Coronary angiography demonstrated a type II SCAD in the mid to distal LAD with no evidence of atherosclerotic disease in other vessels (Figure 1a). Right heart catheterization revealed mildly elevated filling pressures and a cardiac index (CI) of 1.7, consistent with cardiogenic shock. Intra-aortic balloon pump (IABP) was placed to augment cardiac output and perfusion. She was transferred to a quaternary care center for continued cardiac critical care support.

**Figure 2:**
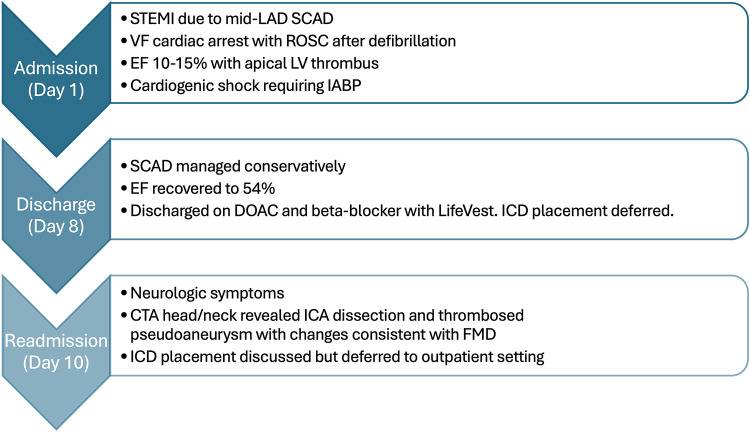
A brief clinical course of the patient from initial presentation with spontaneous coronary artery dissection through readmission with carotid artery dissection clinically consistent with diagnosis of fibromuscular dysplasia.

Interventional cardiology considered revascularization given her hemodynamic instability requiring mechanical circulatory support, but the risks associated with percutaneous coronary intervention (PCI) were deemed prohibitive, including the presence of a large diagonal branch originating near the SCAD site with a risk of propagation, and extensive intramural hematoma, increasing the likelihood of further vessel compromise with intervention. The patient was closely monitored for improvement with medical therapy with a plan for CABG if ischemia worsened.

By hospital day 2, LVEF improved to 35% with a new LV thrombus on TTE, prompting initiation of anticoagulation (Figure 1b). A prospective, coronary computerized tomographic angiography (CTA) using the test-bolus technique with 0.75 mm slices revealed resolution of SCAD. By hospital day 7, IABP was removed. Repeat echocardiography on hospital day 8 showed LVEF normalization to 54%. She was transitioned to metoprolol succinate and dabigatran. Given her history of ventricular arrhythmia, secondary prevention ICD placement was discussed. A wearable defibrillator provided until the patient had outpatient cardiac follow-up within the month while undergoing further workup prior to final decision regarding ICD implantation. The patient was discharged with a plan for outpatient vasculopathy and rheumatologic workup along with dabigatran for three months (chosen for insurance purposes) with planned repeat echocardiography to reassess LV thrombus. Anticoagulation benefit of clearing the ventricular thrombus outweighed the risk of worsening intramural hematoma in the LAD given evidence of SCAD resolution.

Ten days after discharge, the patient returned to the ED, complaining of severe tingling of her scalp and back, along with disequilibrium and emesis. Her ECG remained unchanged from prior to admission. A CTA of the head and neck was ordered to evaluate for possible vascular occlusion, which revealed a dissection with a partially thrombosed pseudoaneurysm of the distal right cervical internal carotid artery (ICA), with approximately 40% stenosis of the true lumen (confirmed on digital subtraction angiography, Figure 1c). Additionally, there was evidence of FMD affecting the right ICA (Figure 1d). Neurosurgery was consulted, and operative management was deferred as there was no flow limitation (Figure 2).

At repeat cardiology visits over the nine months post-discharge, the patient was noted to be well appearing, back to work, and participate actively in daily life. Without any alerts or discharges from her LifeVest, recovery of her ejection fraction and healed LAD, her cardiologist felt it was safe to discontinue the LifeVest. After extensive discussions with the patient, there is no plan for subcutaneous or transvenous ICD implantation. The patient expressed relief at avoiding further invasive therapy and valued shared decision-making in ICD deferral. Her rheumatology team has yet to determine the interval of which she will be routinely imaged for further evidence of FMD.

## Discussion

SCAD accounts for approximately 4% of all ACS cases and up to 35% of ACS cases in women under age 50. It predominantly affects women, with a mean age of 44–53 years. It is a significant cause of pregnancy-associated ACS, comprising up to 40% of acute myocardial infarctions during pregnancy and the postpartum period (1, 3). Emotional stress has been identified as a potential precipitating factor for SCAD in women, while physical stressors have been more commonly reported in men (1, 2).

On angiography, SCAD often appears as smooth narrowing of the coronary artery with distal tapering and tends to affect the LAD (4). Acute management of SCAD is preferentially conservative, as thrombolysis and percutaneous coronary intervention (PCI) are generally not recommended due to concern for dissection propagation or hematoma extension; however, they may be considered in unstable patients. Most SCAD cases recover with conservative management, and repeat angiography often shows spontaneous healing of the affected coronary arteries within 30 days (1).

FMD is found in up to 54% of patients with SCAD. While SCAD and FMD are distinct entities, the presence of FMD increases the likelihood of developing vascular complications in other areas of the body, such as intracranial aneurysms and dissections (1, 5, 6). Given the association between SCAD and FMD, patients with SCAD should undergo imaging screening for FMD, though there is no defined timeline for this screening (1, 7). In this case, the instance of both coronary and carotid dissections within weeks emphasizes the systemic nature of fibromuscular dysplasia, but current prospective data does not note an increased risk of mortality or repeat dissection (8).

Secondary prevention ICD in SCAD remains controversial due to a lack of conclusive evidence supporting its benefit, with very few studies in this population (9–11). The AHA and ESC stress that reversible causes of ischemia, such as SCAD, should not warrant secondary prevention ICD (7, 12). What has yet to be defined is if SCAD due to an underlying predisposition such as FMD is considered “reversible.” Recurrence rates in SCAD are estimated at 10%–30% over long-term follow-up, with higher risk among patients with associated arteriopathies such as FMD (1, 13).

It is understandable that clinicians and patients may feel inclined to consider ICD placement in this population of young patients. It is important to remember that ICD placement carries multiple potential harms, including infection, perforation, pneumothorax, and bleeding, with long-term infectious risks associated with intravascular devices (14). Clinicians must consider that the number of replacements increases the earlier an ICD is placed, with the average lifespan of ICD generators and leads being 5–10 years and 10–15 years respectively (12, 14). Subcutaneous ICDs may reduce intravascular complications compared with transvenous devices (15). In this case, outpatient EP consultation addressed this option, but implantation was deferred after discussion with the patient. Given a lack of clear guidelines, ICD implantation should be guided by an informed risk/benefit discussion.

## Conclusion

This case highlights the complexity of diagnosing and managing SCAD associated with ventricular arrhythmia and cardiac arrest, particularly in the context of FMD. Long-term management of these patients requires serial vascular imaging and multidisciplinary follow-up. This case brings into question whether secondary prevention ICD is warranted and highlights the need for further research in this population.

## Data Availability

The original contributions presented in the study are included in the article/Supplementary Material, further inquiries can be directed to the corresponding author.
